# Association between Trace Elements and Body Composition Parameters in Endurance Runners

**DOI:** 10.3390/ijerph17186563

**Published:** 2020-09-09

**Authors:** Gema Barrientos, Javier Alves, Víctor Toro, María Concepción Robles, Diego Muñoz, Marcos Maynar

**Affiliations:** 1 Department of Sport Science, Faculty of Education, Pontifical University of Salamanca, C/Henry Collet, 52-70, CP: 37007 Salamanca, Spain; gbarrientosvi@upsa.es; 2 Department of Physiology, Faculty of Sports Science Faculty, University of Extremadura, Avda de la Universidad, s/n CP: 10003 Cáceres, Spain; vtororom@alumnos.unex.es (V.T.); mcroblesgil@unex.es (M.C.R.); diegomun@unex.es (D.M.); mmaynar@unex.es (M.M.)

**Keywords:** trace elements, skinfolds, fat mass, muscle mass, bone mass

## Abstract

The aim of this study was to determine the possible correlations between essential and toxic trace elements of plasma with several anthropometric and body composition parameters and performance in endurance runners. Sixty-five high-level middle and long-distance runners (21  ±  3 years; 1.77 ± 0.05 m; 64.97 ± 7.36 kg; VO_2_ max. 67.55 ± 4.11 mL/min/kg) participated in the present study. Abdominal, subscapular, iliac crest, triceps, front thigh and medial calf skinfold thicknesses and an incremental test until exhaustion were recorded. Body, fat, muscle and bone mass were estimated. Plasma trace elements were analyzed with inductively coupled plasma mass spectrometry (ICP-MS). Correlations and simple linear regression were used to assess the relationship between trace elements and several variables. Different skinfolds, fat mass, muscle mass and bone mass correlated positively and negatively with trace elements such as copper, manganese, selenium, vanadium, zinc, lithium, rubidium, strontium, arsenic, beryllium and lead. Lithium was related with performance. In conclusion, endurance training causes changes in the body concentrations of several trace elements that trigger modifications in body composition that may be interesting, if confirmed in the future, for the control of metabolic diseases such as obesity.

## 1. Introduction

Several anthropometric and body composition variables are known to be associated with endurance performance in elite runners [1]: body weight [2], fat and muscle weight [3] as well as different skinfolds [4,5]. Moreover, an excess of fat mass requires greater muscular effort and higher energy expenditure [6] and results in the depletion of glycogen stores, negatively affecting sport performance. Trace elements (TEs) are present in body tissues with different fundamental biological functions such as protein metabolism, bone metabolism and antioxidant function [7]. Endurance training in athletes during long periods results in the mobilization of some TEs into the circulation [8]. Important interactions have been reported between TE concentrations and the antioxidant system, as TE influences antioxidant enzymes and vice versa [9,10]. Toxic TEs such as arsenic (As), cadmium (Cd), lead (Pb) and mercury (Hg) increase reactive oxygen species (ROS) production and can cause oxidative stress (OS) due to the imbalance between oxidants and antioxidants in favor of oxidants, which alters the correct functioning of thyroid hormones and the hypothalamic–pituitary–adrenal (HPA) axis [11]. The HPA axis is a key mediator in the neuroendocrine system, as well as in hormone synthesis and energy metabolism [12].

Physical exercise is another factor that increases ROS production [13]. The accumulation of high loads of daily training in endurance runners increases the possibility of producing OS when ROS levels exceed the capacity of the antioxidant system—a situation that can cause molecular damage [14,15]. Essential TEs such as selenium (Se), zinc (Zn) and copper (Cu) have antioxidant functions; they are also cofactors in important antioxidant enzymes such as glutathione peroxidase (GPH-Px) and zinc–copper superoxide dismutase (Zn-Cu SOD) that participate in the reduction of OS induced by ROS [16]. Current evidence indicates that OS favors the deposition and accumulation of fatty tissue [17], and an essential deficiency of TEs seems to be associated with an increase in the deposition of fat in the organism [18]. The homeostasis of TE in adipose tissue is associated with obesity-related metabolic disorders [19].

To our knowledge, few studies have investigated the interactions between TE concentrations and body composition variables in runners. Therefore, the objective of this study was to determine the plasma concentrations of toxic and essential TEs in high-level endurance runners and to observe the relationships with performance and several anthropometric and body composition variables; furthermore, we aimed to study whether runners with adequate plasma concentrations of essential TEs and lower concentrations of toxic TEs reported less fat mass and lower skinfolds, as well as better sport performance.

## 2. Materials and Methods

The criteria for the selection of participants in our study iwere similar to those proposed in a previous study by Barrientos et al. [20].

### 2.1. Participants

Sixty-five male middle and long-distance runners (21  ±  3 years) recruited from the same area of Spain (Extremadura) participated in the survey. Subjects were included if they had lived in the region for no less than 3 years, had been training continuously for at least 6 years and competed in national and international tournaments (1500 to 5000 m race modalities and cross country), had good results and a personal best in modalities of 3:37.79–4:08.24 for 1500 m and 13:11.01 and 15:10.35 for 5000 m and had taken no TE supplementation for 3 months prior to the study. All subjects participated voluntarily, were informed about the purpose of the study and possible risks and gave their written consent. The study was approved by the Bioethics Committee of the University of Extremadura, and all procedures were performed in accordance with the Declaration of Helsinki regarding human rights, updated at the World Medical Assembly in Seoul in 2008 for research with human subjects (register number 52/2012).

### 2.2. Nutritional Assessment

The nutritional composition of the participants’ diets was determined using different nutrient databases [21,22,23] that were used for information about macronutrients and micronutrients. The athletes completed a 3 day nutritional questionnaire on two working days and one weekend day, where they indicated the amount (in grams) of all food ingested on those days.

### 2.3. Training Characteristics

The runners had performed physical training regularly during the previous 6 years. They trained 6 days a week (some runners did double sessions), recording an average weekly distance between 80–155 km, doing 15–25% above the anaerobic threshold and 75–85% aerobically. The weekly training routines consisted of 3–6 sessions/week of continued aerobic running and 2–4 sessions/week of intense series and interval training. They also performed two weekly sessions of resistance training with high volume (3–5 sets of 8–25 repetitions of whole-body exercises) and low–moderate intensity (30–70% of 1RM) depending on the period of the season.

### 2.4. Anthropometric Measures

Body composition measurements were always recorded at the same time (9:00–10:00 a.m.) after an overnight fast and without intense training or competitions for at least 72 h before the test.

Height was measured with an accuracy of 0.1 cm using a wall-mounted stadiometer (Seca, Hamburg, Germany), and body weight was measured with an accuracy of 0.01 kg using a calibrated electronic digital scale (Seca, Hamburg, Germany) in barefoot conditions. Fat mass content was estimated from the sum of six skinfolds (∑6) (abdominal, suprailiac, triceps, subscapular, front thigh and medial calf) [24]. The thicknesses of the skinfolds were measured with a plicometer (Holtain, Crymych, UK) and recorded to the nearest 0.2mm. The upscale pressure of the caliper was checked according to the manufacturer’s specification. Measurements were taken three times 2 s after the application of the caliper by the same investigator—an expert in kinanthropometry techniques (accredited level 1) who had previously shown a test–retest reliability of r > 0.9. Body composition was calculated according to the indications of the International Society for the Advancement of Kinanthropometry [25].

### 2.5. Physical Performance Evaluation

An incremental test until exhaustion was performed on a treadmill (Sport engineering limited, Powerjog, Birmingham, UK) and an ergospirometer system equipped with a gas analyzer (Metamax, Cortex Biophysik, Gmbh, Leipzig, Germany) to measure the athletes’ VO_2_ max. scores and performance, the validity and reliability of which have been established previously [26]. A Polar pulsometer (Polar Vantage M, Norway) was used to evaluate the maximum heart rate.

After a 10 min warm-up at 8–10 km/h, the athletes initiated the test at a speed of 10 km/h, which increased by 1 km/h every 400 m until voluntary exhaustion. VO_2_ max. was determined with breath-by-breath data according to the following criteria: the respiratory exchange ratio (RER) had to exceed 1, there had to be a plateau in oxygen uptake (VO_2_), an increment in carbon dioxide (CO_2_) elimination and an increment in the ventilatory volume (VE) induced by the increases in the test velocity [27].

### 2.6. Blood Sample

At 9:00 AM, after the weigh-in, 10 mL of venous blood was drawn from the antecubital vein of each participant using a plastic syringe fitted with a stainless-steel needle. The sample was extracted before the exercise test. Once obtained, the samples were stored in a metal-free polypropylene tube with Ethylenediaminetetraacetic acid (EDTA) as the anticoagulant.

Later, the blood samples were centrifuged at 3000 rpm for 10 min at room temperature to isolate the plasma. The plasma was aliquoted into an Eppendorf tube and conserved at −80 °C until biochemical analysis.

### 2.7. Sample Determination

TE analyses were performed using inductively coupled plasma mass spectrometry (ICP-MS) following Maynar et al. [17].

Briefly, to prepare the analysis, the decomposition of the organic matrix was achieved by heating it for 10 h at 90 °C after adding 0.8 mL HNO_3_ and 0.4 mL H_2_O_2_ to 1 mL of plasma sample. The samples were then dried at 200 °C on a hot plate. Sample reconstitution was carried out by adding 0.5 mL of nitric acid, 10 μL of indium (10 mg/L) as an internal standard and ultrapure water to bring the solution to 10 mL. Reagent blanks, element standards and certified reference materials (Seronorm, lot 0511545 Sero AS Billingstand, Norway) were prepared identically and used for accuracy testing. Before the analysis, the commercial control materials were diluted according to the manufacturer’s recommendations. Digested solutions were analyzed by an ICP-MS Nexionmodel 300D (PerkinElmer, Inc., Shelton, CT, USA). Samples were analyzed in triplicate. The values of each standard material (10 μg/L) used for quality controls were in agreement with intra and inter-assay coefficient variations of less than 5%.

### 2.8. Statistical Analysis

The statistical treatment of the results was performed using the SPSS 21.0 statistical program for Windows (IBM Co., Armonk, NY, USA). The results were expressed as mean ± standard deviation (mean ± sd). The normality of the distribution of variables was analyzed using the Kolmogorov–Smirnov tests, and the homogeneity of the variances was analyzed with the Levene test. Simple linear regression analysis was conducted to examine the associations between plasma TE concentrations and several anthropometric and body composition parameters. Pearson’s coefficient of correlation (r), beta coefficient (β) and coefficient of determination (R^2^) were calculated. The strength of the linear relationship was determined according to Chan’s guidelines [28]; a value of at least 0.8 represented a very strong relationship, 0.6–0.8 showed a moderately strong relationship, 0.3–0.6 showed a fair relationship, and a value of less than less than 0.3 indicated a poor relationship. A *p*-value < 0.05 was considered to be statistically significant.

## 3. Results

The results obtained in our study are detailed below. Table 1 shows the anthropometric, body composition characteristics and performance values of the runners, along with VO_2_ max. scores (68.12 ± 4.21 mL/min/kg) and the total meters (m) that the athletes ran in the incremental test (4579.94 ± 535.81 m).

The intake of the different TEs consumed in the diet is shown in Table 2.

Table 3 shows the plasma concentrations of essential and toxic TEs, respectively. Plasma values of Cu were lower (750 ug/L) than the published range; other plasma TE values analyzed were comparable to the reference values.

Table 4, Table 5 and Table 6 illustrate the different correlations found between the plasma TE concentrations and several anthropometric, body composition and performance parameters found in the runners.

Cu had a negative correlation (r = −0.342; β = −0.004; *p* = 0.017) with the subscapular skinfold, fat mass (r = −0.296; β = −0.002; *p* = 0.041), muscle mass (r = −0.295; β = −0.009 *p* = 0.042) and bone mass (r = −0.359; β = −0.003; *p* = 0.012).

Manganese (Mn) presented positive correlations with abdominal (r = 0.348; β = 0.634; *p* = 0.015) and suprailiac skinfolds (r = 0.315; β = 0.340; *p* = 0.029) and negative correlations with the medial calf skinfold (r = −0.376; β = −0.562; *p* = 0.008).

Plasma Se showed a negative correlation with fat mass (r = −0.470; β = −0.029; *p* = 0.006), abdominal (r = −0.304; β = −0.061; *p* = 0.035) and subscapular skinfolds (r = −0.368; β = −0.047; *p* = 0.010) and the sum of six skinfolds (r = −0.337; β = −0.231; *p* = 0.019).

As for plasma vanadium (V), a negative correlation was found with the medial calf skinfold (r = −0.405; β = −2.518; *p* = 0.004).

Zn presented a negative relationship with the abdominal skinfold (r = −0.377; β = −0.007; *p* = 0.008).

Lithium (Li) concentrations showed a negative relationship with the abdominal (r = −0.323; β = −1.094; *p* = 0.025) and subscapular skinfolds (r = −0.387; β = −0.823 *p* = 0.007) and the sum of six skinfolds (r = −0.322; β = −3.694; *p* = 0.026), as well as fat mass (r = −0.407; β = −0.505; *p* = 0.004), muscle mass (r = −0.355; β = −1.725; *p* = 0.013) and bone mass (r = −0.369; β = −0.527; *p* = 0.010), and a positive relationship with total meters run (r = 0.368; β = 243.53; *p* = 0.010).

Regarding rubidium (Rb), we found negative correlations with fat mass (r = −0.363; β = −0.016; *p* = 0.011), muscle mass (r = −0.386; β = −0.067; *p* = 0.007), bone mass (r = −0.532; β = −0.027; *p* = 0.001) and the subscapular skinfold (r = −0.532; β = −0.024; *p* = 0.000).

In the plasma concentrations of strontium (Sr), negative correlations were found with the abdominal (r = −0.309; β = −0.105; *p* = 0.033) and tricipital skinfolds (r = −0.335; β = −0.073; *p* = 0.005), and a positive relationship with muscle mass (r = 0.362; β = 0.178; *p* = 0.011).

In relation to toxic TE, As presented an inverse relationship with the thigh skinfold (r = −0.311; β = −0.335; *p* = 0.032). Beryllium (Be) showed a negative correlation with the medial calf skinfold (r = −0.346; β = −22.806; *p* = 0.016) and a positive correlation with muscle mass (r = 0.303; β = 34.972; *p* = 0.036). Finally, we found a positive correlation between Pb concentrations and the abdominal skinfold (r = 0.300; β = 0.786; *p* = 0.038).

## 4. Discussion

In the present study, middle–long-distance runners presented body composition values comparable to those reported in subjects with the same characteristics, with low fat mass and skinfold values, as has been reported previously, as excess fat mass adversely affects performance [1]. The variability of several anthropometric and body composition parameters is a consequence of the different training degrees of the runners, as they periodize their training and body composition [29], because maintaining a low fat mass and skinfolds for prolonged periods could negatively affect their health. The runners had high VO_2_ max. values (67.55 ± 4.11 mL/min/kg)—a parameter required in high-level endurance athletes [5].

In the runners, average dietary TE were adequate to comply with the Dietary Reference Intakes (DRI) in adults. The plasma TE values analyzed in this study can be considered to be comparable to the reference values previously reported, except for Cu [21]. In our athletes, we found hypocupremia, and plasma values of Cu were lower than the published range in another study [30], which could have been caused by the utilization of Cu for the synthesis of ceruloplasmin—an enzyme that plays an essential role in the absorption and release of iron from the liver [31]. In addition, Koury et al. [32] reported that Cu is a cofactor in the copper–zinc superoxide dismutase (Cu-Zn SOD) antioxidant enzyme that acts to reduce the toxicity of ROS and has been shown to increase in humans after long physical activity.

In this study, Cu was negatively related to muscle and bone weight, where 63% of the total body content is stored. As previously mentioned, this element is necessary for the synthesis of Cu-Zn SOD, and its deficit could increase muscle damage as a result of OS. Regarding bone mass, it has been reported that a Cu deficiency reduces bone mineralization [33]. This mineral is a cofactor in the enzyme lysyl oxidase, which strengthens collagen and bone fibers [34]. We also found a negative correlation with the subscapular skinfold and fat mass. Recently, Cu has emerged as an important modifier of body lipids; a deficiency of Cu causes the inhibition of semicarbazide-sensitive amine oxidase (SSAO) activity—a regulatory enzyme of energy utilization processes in adipocytes that favors adipocyte hypertrophy and fat deposition [35]. It is thus necessary to check this TE in runners, because excess fat mass is negatively related to sport performance [1]

Li plays an important role in several physiological functions—neural communication, metabolism and cell proliferation—but a deficit or excess concentrations induce negative or toxic effects [36]. The plasma Li concentrations of our runners were lower than the ranges established in adults—from 7 to 28 ug/L [37]. The athletes presented a negative correlation between the Li and abdominal and subscapular skinfolds, as well as the sum of four and six skinfolds and fat mass. A recent study by Castillo-Quan et al. [38] reported that Li is an inhibitor of glycogen synthase kinase 3 (GSK-3), an enzyme that regulates glycogen levels in tissues and can reduce triglyceride concentrations in flies, even when they feed on high-carbohydrate diets, which would favor a lower accumulation of fat mass in runners due to the greater use of fat as an energy substrate in the training sessions. In addition, several studies have reported that, at low doses, Li protects cells against death caused by OS [38,39], and it has been reported that OS favors the deposition and accumulation of fat mass [17].

We also found a negative relationship between Li and bone weight, and it has been suggested that Li may act as a substitute for potassium (K) and magnesium (Mg) and replace them at sites not occupied by them [36]. Tucker et al. [40] reported that both minerals contribute to maintaining adequate bone mineral density; therefore, Li could be used in runners to replace K and Mg in bone remodeling, since their bones support the greater stress caused by the impact of running in the training sessions. A negative correlation was obtained for Li and muscle mass. Lithium is an element with antioxidant functions in adequate concentrations [39]; however, it has a toxic effect in high concentrations [36]. It has been suggested that Li has the ability to interfere in cellular processes such as the redox state and is able to produce damage to DNA and tissues [41]. Therefore, high Li plasma concentrations would induce muscle catabolism as a result of OS in the runners.

Finally, a positive relationship was found between Li and performance, as the runners with the highest plasma Li concentrations ran more meters in the incremental stress test, which could be related to the antioxidant function of Li as well as its action on runners’ glycogen metabolism [38]. As reported, Li inhibits the activity of the GSK-3 enzyme [38]. We hypothesized that this performance improvement could be the consequence of an optimization in fat metabolism of the runners, delaying the use of glycogen as an energy substrate for higher running intensities. Li is an element that generates controversy given the current knowledge and available data, and therefore its possible ergogenic and other effects should be studied more extensively.

Mn is an essential metal required for proper immune function, the regulation of cellular energy, reproduction and bone growth, and it acts as cofactor in manganese superoxide dismutase (Mn-SOD)—a mitochondrial antioxidant enzyme which neutralizes ROS [42]. This TE presented a positive correlation with the abdominal and suprailiac skinfolds and a negative correlation with the medial calf skinfold. It is known that physical activity generates an increase in ROS production and OS [43] that favors fat mass deposition. Research has concluded that adipose tissue increases the local and systemic release of proinflammatory adipocytokines, and this low-grade chronic inflammatory response increases ROS production and permanent OS, with adverse effects on health [44]. There would be an attempt to reverse these inflammatory responses by the action of Mn-SOD when fat tissue is accumulated at the central level in the body, since visceral fat has a more negative impact on health [45].

Se is an essential mineral that participates in the metabolism of thyroid hormones through the iodothyronine deiodinases and is a cofactor of glutathione peroxidase (GPH-Px) and other selenoproteins [46]. Plasma Se concentration had an inverse correlation with fat mass, abdominal and subscapular skinfolds and the sum of four and six skinfolds. Several studies have shown the relationship between subjects with low plasma Se concentrations and obesity, as a result of a higher inflammatory state that increases OS and reduces their concentrations for use as an antioxidant by different selenoproteins [47]. As mentioned, intensive training generates an increase in ROS production, and an augmented production of GPH-Px and the synthesis of other selenoproteins in the athletes would occur to protect against OS, decreasing plasma Se concentrations. It has also been suggested that Se might have the ability to inhibit adipocyte hypertrophy, the accumulation of cholesterol and abdominal fat as a consequence of the regulation of the gene for the β-oxidation of fatty acids [48]. This element could be used as a treatment in overweight people through the reduction of lipid metabolism; therefore, more studies in humans are necessary to check the possible ergogenic effects of Se and determine the appropriate amount without causing possible harmful effects. We also found an inverse relationship with bone weight; as reported, Se participates indirectly in bone health, and Chinese populations with low plasma levels reported more bone disorders as a result of Kashin–Beck disease, which induces necrosis in bone growth cartilage chondrocytes [34].

Plasma V concentration is inversely related to the medial calf skinfold. Previous studies have related this skinfold with performance in endurance runners, because with a lower body mass concentrated in the legs, they would perform less work during running, if other factors were unchanged [49]. V is a TE that is attributed mimetic properties to insulin (I); both V and I can increase adiponectin content in adipocytes [34]. Adiponectin is secreted by adipocytes and stimulates fatty acid oxidation, reduces plasma triglycerides and improves glucose metabolism by increasing insulin sensitivity [50]. It has been hypothesized that some compounds of V could be used as substitutes for insulin in diseases such as diabetes; however, the latest reviews do not support this, since long-term treatment could cause toxicity due to the possible excess accumulation of V [51].

The concentration of Zn in plasma had an inverse correlation with the abdominal skinfold. In adult subjects, low zinc concentrations were associated with increased abdominal fat [52]. Recent scientific evidence seems to indicate that Zn acts as an important regulator of homeostasis of zinc-α2-glycoprotein (ZAG); this adipokine plays an essential role in lipid mobilization and glucose homeostasis regulation. Deficiencies of this essential mineral in the athletes could compromise the physiological functions of ZAG [53]. Balaz et al. [54] indicated that subcutaneous fatty tissue is the main producer of ZAG, and its concentrations in obese subjects were notably reduced, showing a potential role of this adipokine in protecting against the development of obesity, although more studies are needed to clarify the relationship between Zn and ZAG metabolism in obesity.

Regarding plasma Rb concentrations, we found inverse correlations with fat, muscle and bone mass. Rb affects the activity of Na^+^ and K^+^-ATPase due to the similar physicochemical property of Rb to potassium (K); this enzyme plays an important role in the homeostasis of Na^+^, K^+^ and Ca^2+^ in muscle cells and in the excitability of their membrane, contributing to the maintenance of vascular tone [55]. Stolk et al. [56] suggested that Rb released larger amounts of neuronally stored norepinephrine to central adrenergic receptors, the known effects of which would cause an increase in heart rate, increased thermogenesis of brown adipose tissue, increased glucose uptake in the muscle as well as an increase in fatty tissue lipolysis that would favor the loss of fat mass in runners. With respect to muscle mass, we hypothesize that an excess increase in energy expenditure could cause an energy deficit in athletes that would induce catabolism and the loss of muscle weight. In relation to bone mass, it has recently been reported that K is positively related to better bone mineral density [57]; as mentioned, Rb can replace the functions of K [55] and therefore would be used by the body to maintain adequate bone health in athletes.

Sr is a mineral that is stored in bones and is thought to have a double action: promoting osteogenic bone formation and inhibiting osteoclastic bone resorption [58]. In relation to Sr, we found a positive relationship with muscle mass, Gulhan et al. [59] reported a study in osteoporotic women supplemented with Sr, where the concentrations of insulin-like growth factor 1 (IGF-1), an anabolic hormone that promotes muscle growth, increased significantly. In addition, IGF-1 participates in the metabolism of lipids and glucose, favoring fat oxidation and possibly benefitting fat mass loss [60], as occurred in our study, since there is a negative relationship of this element with the tricipital and abdominal skinfolds. However, more studies are necessary to understand and determine these functions of Sr.

We found an inverse relationship between plasma As concentrations and the thigh skinfold, although with the current scientific evidence, we cannot justify this relationship. We hypothesize that fat weight could store As, and when runners lose fat mass, this toxic TE is eliminated.

Be plasma concentrations had a positive relation with muscle mass. The lung is the main Be storage place in the organism [21], although we hypothesize that muscle weight is also a storage place. Maynar-Marino et al. [61] reported that athletes of different sport modalities had more Be concentrations and more muscle mass than control subjects.

In the present study, we found a positive relationship between plasma Pb concentrations and the abdominal skinfold. Different studies have reported that Pb is a TE that favors the production of ROS, and it is considered to be an endocrine disruptor with possible obesogenic effects, favoring the increase and accumulation of fatty tissue [11,62], which would be negative for performance. Several studies have reported that Se is an essential element to combat oxidative stress induced by Pb due to its antioxidant function [63], and it is necessary to check the intake and consider possible supplementation of this element.

Limitations of this study included the fact that TEs enter the body by different paths; one of the main exposure sources is diet. In this survey, nutritional intake was analyzed by recording the number of grams of each food ingested during the evaluation days; the amount of each specific mineral was obtained using several databases. The intake of TE by the runners was obtained using a self-report questionnaire that introduced some inaccuracies. It should be borne in mind that the quantity of TE which we find in the same foods is variable depending on its origin (environmental characteristics, type of soil, etc.) as well as the possible interactions that occur as a result of their preparation, consumption and metabolism. The use of dual-energy X-ray absorptiometry (DXA) or bioelectrical impedance analysis (BIA) methods could provide more accurate data on the body composition of subjects. Another limitation was that some of the studies that have been cited in the discussion used different methodologies. Likewise, although we have used the most significant correlations, some of our assessments may be somewhat speculative, due to the scarcity of studies in humans—and specifically in runners—and may not allow us to be able to present a robust discussion. Future studies are required to clarify these points.

## 5. Conclusions

Endurance training causes changes in the body concentrations of several trace elements that can trigger modifications in body composition. Thus, endurance runners should check the intake of essential TEs such as Cu, Se, V and Li in order to avoid any possible cases of deficit and establish optimal supplementation regimens. We must encourage endurance runners to follow diets with foods rich in these minerals to avoid possible deficits as a consequence of their continuous training loads. This could protect them against excess ROS production and exercise-induced OS and thus could facilitate fat deposition and possible changes in body composition.

More studies are required with endurance runners to clarify the possible relationships found between the trace element concentrations analyzed and the changes in body composition; these relationships may be of future interest in widely extended pathologies such as obesity.

## Figures and Tables

**Table 1:** Anthropometric, body composition and performance values in the runners.

Parameters	Runners	Ranges
Body mass (kg)	64.97 ± 7.36	49.7–78.1
Fat mass (kg)	5.35 ± 1.01	3.45–7.87
Bone mass (kg)	11.91 ± 1.15	11.90–13.86
Muscle mass (kg)	32.02 ± 3.96	22.65–38.15
Abdominal skinfold (mm)	9.43 ± 2.75	5–16.8
Suprailiac skinfold (mm)	6.22 ± 1.63	3.8–12.5
Subscapular skinfold (mm)	8.43 ± 1.73	5.2–14.6
Triceps skinfold (mm)	6.42 ± 1.76	3.8–11.3
Front thigh skinfold (mm)	9.04 ± 2.90	5.2–15.8
Medial calf skinfold (mm)	7.44 ± 2.26	3.3–13.4
∑6 skinfolds (mm)	46.97 ± 9.34	32.4–73.7
Distance (m)	4584.04 ± 537.84	3988–5619
VO_2_ max. (mL/min/kg)	68.12 ± 4.21	55.15–77.03
Maximum heart rate (bpm)	192.1 ± 6.75	186.2–198.4

VO_2_ max: maximum oxygen uptake; ∑6 skinfolds: sum of six skinfolds.

**Table 2:** Food intake of trace elements in the runners.

Trace Elements, Recommended Intake	Intake
As (12–300 mg/d)	1689 ± 834.15
B (0.75–1.35 mg/d)	1.33 ± 1.50
Be (<50 µg/d)	9.69 ± 8.99
Cd (<70 µg/d)	23.34 ± 15.40
Co (200–300 µg/d)	296.01 ± 214.97
Cu (2000–3000 µg/d)	1675.96 ± 566.91
Li (180–550 µg/d)	366.95 ± 396.90
Mn (2500–5000 µg/d)	3381.40 ± 1439.97
Mo (75–400 µg/d)	308.99 ± 181.93
Pb (<400 µg/d)	209.10 ± 142.78
Rb (1.5–7 mg/d)	3.904 ± 4.785
Se (50–200 µg/d)	76.42 ± 44.99
Sr (1000–2300 µg/d)	1890.76 ± 1784.30
V (10–70 µg/d)	25.48 ± 29.28
Zn (10–15 mg/d)	11.10 ± 3.72

Co: cobalt; Cu: copper; Mn: manganese; Mo: molybdenum; Se: selenium; V: vanadium; Zn: zinc; B: boron; Li: lithium; As: arsenic; Be: beryllium; Cd: cadmium; Pb: lead; Rb: rubidium; Sr: strontium.

**Table 3:** Plasma concentrations of trace elements in the runners.

Trace Elements	Runners	Range
As (µg/L)	2.34 ± 2.80	0.24–12.00
B (µg/L)	8.65 ± 10.98	0–59.98
Be (µg/L)	0.07 ± 0.03	0–0.15
Cd (µg/L)	0.07 ± 0.04	0.02–0.23
Co (µg/L)	0.68 ± 0.11	0.47–0.87
Cu (µg/L)	692.16 ± 132.53	453.9–937.01
Li (µg/L)	1.38 ± 0.79	0.35–4.75
Mn (µg/L)	2.05 ± 1.49	0.21–5.48
Mo (µg/L)	0.63 ± 0.60	0.11–3.35
Pb (µg/L)	0.96 ± 1.08	0.01–4.93
Rb (µg/L)	138.50 ± 23.01	98.79–186.01
Se (µg/L)	96.56 ± 13.89	69.75–124.10
Sr (µg/L)	26.23 ± 8.12	14.79–47.50
V (µg/L)	0.30 ± 0.37	0–1.80
Zn (µg/L)	791.90 ± 144.01	538.02–1208.95
As (µg/L)	2.34 ± 2.80	0.24–12.0

As: arsenic; B: boron; Be: beryllium; Cd: cadmium; Co: cobalt; Cu: copper; Li: lithium; Mn: manganese; Mo: molybdenum; Pb: lead; Rb: rubidium; Se: selenium; Sr: strontium; V: vanadium; Zn: zinc.

**Table 4:** Correlations between abdominal, suprailiac, subscapular and triceps skinfolds with trace elements in the runners.

TE	Abdominal (mm)				Suprailiac (mm)				Subscapular (mm)				Triceps (mm)			
	r	β (95% CI)	R^2^	*p*	r	β (95% CI)	R^2^	*p*	r	β (95% CI)	R^2^	*p*	r	β (95% CI)	R^2^	*p*
Cu									−0.342	−0.004 (−0.01/0.00)	0.117	0.017				
Mn	0.348	0.634 (0.12/1.14)	0.121	0.015	0.315	0.340 (0.03/0.64)	0.099	0.029								
Se	−0.304	−0.061 (−0.11/0.00)	0.093	0.035					−0.368	−0.047 (−0.08/−0.01)	0.125	0.010				
V																
Zn	−0.377	−0.007 (−0.01/0.00)	0.142	0.008												
Li	−0.323	−1.094 (−2.04/−0.14)	0.104	0.025					−0.387	−0.823 (−1.40/−0.24)	0.149	0.007				
Rb									−0.317	−0.024 (−0.04/0.00)	0.100	0.028				
Sr	−0.309	−0.105 (−0.20/−0.01)	0.095	0.033									−0.335	−0.073 (−0.13/−0.01)	0.112	0.020
As																
Be																
Pb	0.300	0.786 (0.04/1.52)	0.090	0.038												

TE: trace elements; Cu: copper; Mn: manganese; Se: selenium; V: vanadium; Zn: zinc; Li: lithium; Rb: rubidium; Sr: strontium; As: arsenic; Be: beryllium; Pb: lead. r: Pearson’s coefficient of correlation; β: beta coefficient; CI: confidence interval; R^2^: coefficient of determination; *p*: *p*-value.

**Table 5:** Correlations between front thigh and medial calf skinfolds and the sum of six skinfolds, as well as the total meters in the incremental test with trace elements in the runners.

TE	Front Thigh (mm)				Medial Calf (mm)				Sum of Six Skinfolds (mm)				Performance (Total Meters)			
	r	β (95% CI)	R^2^	*p*	r	β (95% CI)	R^2^	*p*	r	β (95% CI)	R^2^	*p*	r	β (95% CI)	R^2^	*p*
Cu																
Mn					−0.376	−0.562 (−0.97/−0.15)	0.142	0.008								
Se									−0.337	−0.231 (−0.42/−0.04)	0.114	0.019				
V					−0.405	−2.518 (−4.20/−0.83)	0.164	0.004								
Zn																
Li									−0.322	−3.694 (−6.91/−0.46)	0.104	0.026	0.368	243.53 (61.18/425.87)	0.136	0.010
Rb																
Sr																
As	−0.311	−0.335 (−0.63/−0.03)	0.097	0.032												
Be					−0.346	−22.806 (−41.15/−4.46)	0.120	0.016								
Pb																

TE: trace elements; Cu: copper; Mn: manganese; Se: selenium; V: vanadium; Zn: zinc; Li: lithium; Rb: rubidium; Sr: strontium; As: arsenic; Be: beryllium; Pb: lead. r: Pearson’s coefficient of correlation; β: beta coefficient; CI: confidence interval; R^2^: coefficient of determination; *p*: *p*-value.

**Table 6:** Correlations between fat, muscle and bone mass with mineral trace elements in the runners.

TE	Fat Mass (kg)				Muscle Mass (kg)				Bone Mass (kg)			
	r	β (95% CI)	R^2^	*p*	r	β (95% CI)	R^2^	*p*	r	β (95% CI)	R^2^	*p*
Cu	−0.296	−0.002 (−0.01/0.00)	0.088	0.041	−0.295	−0.009 (−0.01/0.00)	0.087	0.042	−0.359	−0.003 (−0.01/0.00)	0.129	0.012
Mn												
Se	−0.389	−0.029 (−0.05/−0.01)	0.151	0.006					−0.401	−0.034 (−0.05/−0.01)	0.161	0.005
V												
Zn												
Li	−0.407	−0.505 (−0.84/−0.16)	0.165	0.004	−0.355	−1.725 (−3.07/−0.37)	0.126	0.013	−0.369	−0.527 (−0.92/−0.13)	0.137	0.010
Rb	−0.363	−0.016 (−0.03/0.00)	0.132	0.011	−0.386	−0.067 (−0.11/−0.02)	0.149	0.007	−0.532	−0.027 (−0.04/−0.01)	0.268	0.001
Sr					0.362	0.178 (0.04/0.31)	0.131	0.011				
As												
Be					0.303	34.972 (2.32/67.62)	0.092	0.036				
Pb												

TE: trace elements; Cu: copper; Mn: manganese; Se: selenium; V: vanadium; Zn: zinc; Li: lithium; Rb: rubidium; Sr: strontium; As: arsenic; Be: beryllium; Pb: lead. r: Pearson’s coefficient of correlation; β: beta coefficient; CI: confidence interval; R2: coefficient of determination; *p*: *p*-value.
